# Infantile fibrosarcoma of the left upper limb mimicking a hemangioma: A case report

**DOI:** 10.1016/j.ijscr.2025.111417

**Published:** 2025-05-09

**Authors:** Hafedh Daly, Faiez Boughanmi, Mohamed Zayati, Leith Limayem, Mohamed Ali Chaouch, Fethi Jebali

**Affiliations:** a Department of Cardiovascular Surgery, Monastir University Hospital, Tunisia; b Department of General Surgery, Monastir University Hospital, Tunisia; c Department of Anesthesiology and Intensive Care B, Monastir University Hospital, Tunisia

**Keywords:** Case report, infantile fibrosarcoma, Left upper limb, Hemangioma

## Abstract

**Introduction and importance:**

Infantile fibrosarcoma (IFS) is a rare soft tissue malignancy that primarily affects children under one year of age. Its clinical and radiologic features often resemble benign vascular tumors like hemangiomas, making diagnosis challenging. Early and accurate identification is essential for effective management.

**Case presentation:**

We report a case of a 3-year-old child with a progressively enlarging mass in the left upper limb. Clinical examination revealed a firm, non-pulsatile mass with prominent venous collateral circulation. Doppler ultrasound and MRI findings suggested an infantile hemangioma. Surgical excision revealed a highly vascularized tumor closely associated with the brachial artery and median nerve. Histopathological analysis confirmed infantile fibrosarcoma, characterized by spindle-shaped cells with mild atypia, high mitotic activity, hemosiderin deposits, and significant vascular proliferation. Immunohistochemistry was negative for myogenin, cytokeratin, desmin, CD68, and TLE1.

**Clinical discussion:**

This case underscores the diagnostic challenge posed by IFS, which can closely mimic infantile hemangioma on imaging. MRI findings suggested but did not confirm the diagnosis. Histopathology remains the definitive method for diagnosis. Multidisciplinary management—including surgical resection and, when indicated, chemotherapy—is essential for optimal outcomes.

**Conclusion:**

IFS should be considered in the differential diagnosis of congenital soft tissue masses, particularly when atypical features are present. Definitive diagnosis relies on histopathological and immunohistochemical evaluation. Multidisciplinary management plays a critical role in ensuring favorable clinical outcomes.

## Highlights

• The case illustrates the diagnostic difficulty between infantile fibrosarcoma and hemangioma due to overlapping clinical and radiological features. Despite initial imaging suggesting an infantile hemangioma, histopathological and immunohistochemical analysis confirmed the diagnosis of infantile fibrosarcoma.
• The tumor, located in the biceps brachii muscle and intimately associated with the brachial artery and median nerve, was successfully excised through a longitudinal surgical approach without complications.
• The tumor showed spindle-shaped cells with mild atypia, significant mitotic activity (12 mitoses per 10 high-power fields), hemosiderin deposits, chronic inflammatory infiltration, and vascular proliferation with thrombosis and necrotic changes. Immunohistochemical markers were negative for myogenin, cytokeratin, desmin, CD68, and TLE1, supporting the diagnosis of infantile fibrosarcoma.

## 1 Introduction

Hemangiomas, also called childhood hemangiomas or infantile hemangiomas, are the most common benign tumors in childhood [1,2]. There are different types of hemangiomas. Congenital hemangiomas are visible at birth, whereas infantile hemangiomas appear later in infancy. Infantile hemangiomas are characterized by rapid and early growth followed by spontaneous involution [2]. They can pose a problem for the differential diagnosis with congenital malignancies such as the congenital form of infantile fibrosarcoma. The latter has an incidence of 24.5 % of all soft tissue sarcomas observed during the first year of life [3]. It often presents as a painless and poorly circumscribed mass that varies in size and consistency [2]. Its main location is in the extremities, especially in the lower extremities [3]. The treatment of infantile fibrosarcomas is essentially surgical. The use of chemotherapy or radiation therapy is necessary under certain conditions [4]. We report this case, according to SCARE guidelines [5], which shows the diagnostic difficulties that can exist between a hemangioma and an infantile fibrosarcoma.

## 2 Case presentation

**Fig. 1:**
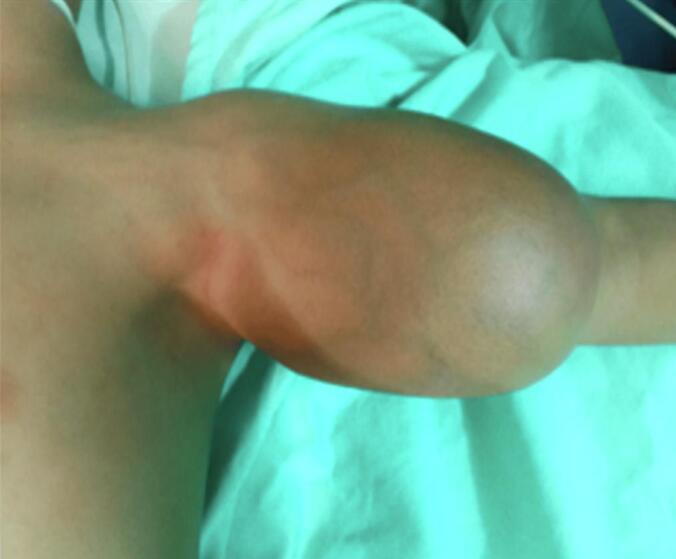
Swelling of the left arm with venous collateral circulation.

**Fig. 2:**
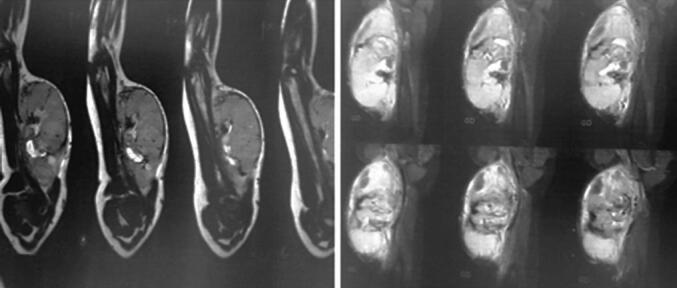
MRI of the left upper limb. T2-magnetic resonance imaging of the infantile fibrosarcoma demonstrating an ovoid mass with moderate hypersignal, traversed by vascular structures (a), which enhances in an intense and homogeneous manner after injection of gadolinium (b).

**Fig. 3:**
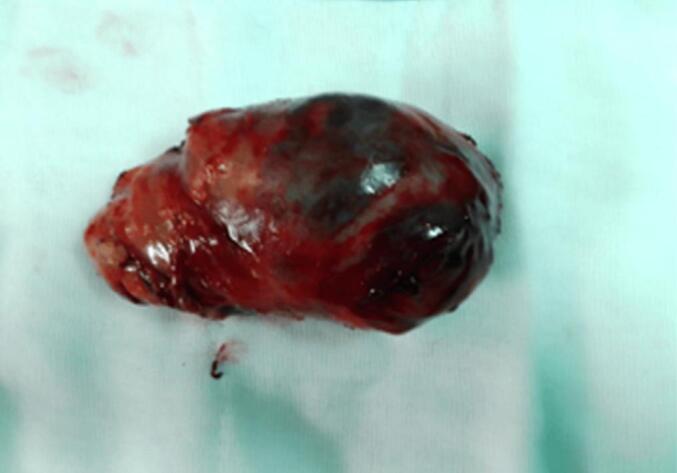
Macroscopic characteristics of the tumor after removal.

**Fig. 4:**
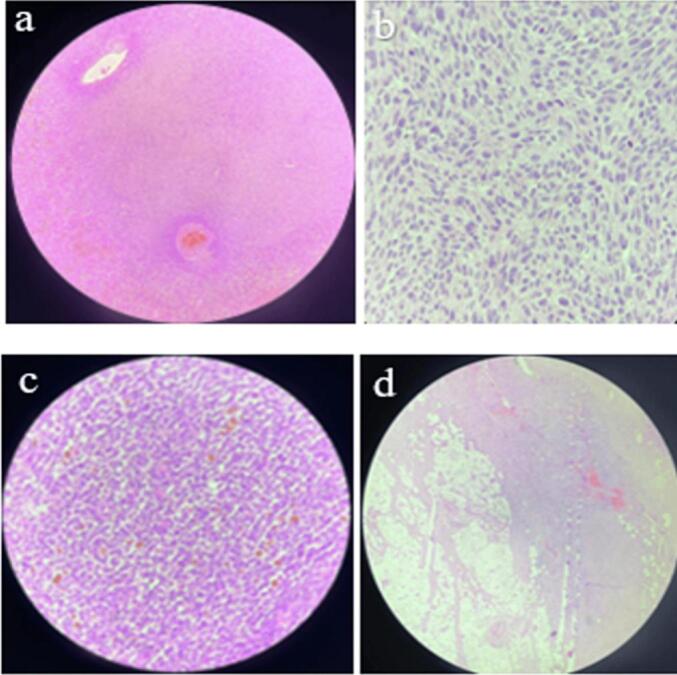
Histopathological appearance of infantile fibrosarcoma. A richly vascularized mesenchymal proliferation (a), made of spindle cells resembling fibroblasts and presenting slight atypia and significant mitotic activity (b) the tumor tissue contained deposits of hemosiderin and dotted with chronic inflammatory infiltrate, composed of lymphocytes, plasma cells and some histiocytes (c). The tumor infiltrated the surrounding adipose and connective tissue (d).

A child aged 3 years and 4 months, with a history of normochromic normocytic no regenerative anemia, was admitted to our department for a large swelling in the left arm. This mass was first observed at age 3 and quickly increased in size. On physical examination, the patient presented a firm, non-pulsatile mass measuring 10 cm in the long axis with the presence of a very developed venous collateral circulation (Fig. 1). Doppler ultrasound revealed a well-circumscribed heterogeneous solid cystic oval mass with a predominant solid component and richly vascularized. It develops in the biceps brachii muscle and has a rich mix of low-resistance arterial and venous vascularization. Small venous lakes with slow circulation are present. This appearance suggests hemangioma. Magnetic resonance imaging revealed a mass at the expense of the biceps brachii muscle, oval, well defined, in the isosignal T 1 and moderate T 2 hypersignal, traversed by hypo intense septa, creating flow voids within it as well as tortuous structures in hypersignal T 1 and T 2 without drop in fat saturation sequences. It presents a homogeneous and intense contrast enhancement after gadolinium injection measuring 8.5 × 5 × 5.8 cm. This aspect favors a vascular tumor at the expense of infantile hemangioma of the biceps brachii muscle (Fig. 2). The patient was operated on via a longitudinal approach to the left arm. It was a richly vascularized tumor, located between the two bundles of the biceps brachii muscle. It is well-limited, has intimate contact with the brachial artery, and is close to the median nerve. Careful dissection allowed for complete excision of this mass. The postoperative course was simple. Macroscopic pathological examination revealed a well-defined, fleshy tumor mass with firm and soft areas, measuring 10 × 6 × 5 cm (Fig. 3). In section, this mass has a multinodular appearance, a beige-yellowish color, sometimes brownish-gray, and presents necrotic-hemorrhagic foci. Microscopically (Fig. 4), the tumor corresponded to moderate and focally high cellularity. This proliferation was made of spindle-shaped cells that resembled fibroblasts and exhibited mild atypia and significant mitotic activity. A maximum of 12 mitoses per 10 fields at high magnification. These cells were arranged in short bundles that intersected in places. In some territories, the tumor was composed of plump cells with ovoid nuclei, without any pararchitecture here was no other detectable cellular differentiation. The tumor tissue contained hemosiderin deposits. It was dotted with chronic inflammatory infiltration, composed of lymphocytes, plasma cells, and a few histiocytes. This proliferation was rich in blood vessels with a wide lumen and walls that were sometimes thin and sometimes quite thick. Vascular thromboses were observed near them and necrotic hemorrhagic and edematous changes. The tumor infiltrated the surrounding adipose tissue and connective tissue (muscular aponeurosis). Immunohistochemical stains were negative for myogenin, cytokeratin, desmin, CD68, and TLE1. Based on these results, the diagnosis of infantile fibrosarcoma was made.

## 3 Clinical discussion

The retained diagnosis was that of infantile fibrosarcoma, but the clinical and radiological aspects initially led to a discussion of infantile hemangioma. This similarity is exceptional. Indeed, Hu Z et al. reported in a review of the literature that only 13 cases of infantile and congenital fibrosarcomas have been reported that presented as a cutaneous vascular tumor, essentially an infantile hemangioma [6]. Some characteristics of congenital fibrosarcoma can be similar to that of infantile hemangioma: it frequently develops in the newborn, usually grows rapidly, may have a vascular appearance, sometimes hemorrhagic fibrosarcoma, and can produce consumptive coagulopathy [7]. Clinically, hemangiomas tend to be softer than fibrosarcomas, which tend to be firm. Fibrosarcomas are often spherical and protruding, sometimes with a grotesque appearance, while hemangiomas tend to be more plaque-shaped, with lobular margins [7]. An unusual appearance or development should alert the physician. Thus, infantile hemangiomas regress with age [8]. Ultrasound of infantile hemangiomas shows a well-defined mass with high vascular density, without abnormality of the surrounding fat, with uniform, pulsatile, and fast flow vascularity in Doppler with arterial and venous waveforms [9]. Magnetic resonance imaging can be used to provide additional information on the extent of local tissue involvement in deep disease. Infantile hemangiomas are hyperintense on T2 and isointense on T1, with intense post-contrast enhancement on MRI. In our case, Doppler ultrasound and MRI findings… were consistent with infantile hemangioma [10]. Fibrosarcomas appear on ultrasound as a homogeneous and well-demarcated mass, but heterogeneity is quite common, consisting of hemorrhagic foci or, more rarely, cystic components. On MRI, they have an isointense T1 and a hyperintense T2 signal intensity with heterogeneous enhancement [11]. When dilated vessels and bleeding are prominent, the tumor may resemble a vascular tumor. Eleti et al. demonstrated in their two-center retrospective study that infantile fibrosarcoma is always highly cellular on magnetic resonance imaging but does not show other specific imaging features. It should be considered in the differential diagnosis of any solid hypertrophied soft tissue mass appearing in the limbs or neck at birth or in infancy [12]. Histologically, fibrosarcoma is characterized by a proliferation of spindle-shaped cells, or atypical cells, in swirling bundles with numerous mitoses, few collagens, and significant vascular proliferation with vascular lacunae without endothelium. The tumor tends to destroy healthy peripheral tissues and develops rapidly. Pulmonary and sometimes lymph node metastases can occur [13]. Treatment is multidisciplinary. Surgery remains the mainstay of treatment [14]. Chemotherapy has a definite role in the neoadjuvant setting. Due to the large size of these tumors that occur in infants and children in sites such as the limbs, with often intimate relationships with adjacent vessels, nerves, or bone, chemotherapy can allow more conservative resection by delayed surgery [15]. In our case, the department of child was referred to the medical oncology to discuss the indication for chemotherapy.

## 4 Conclusions

Infantile fibrosarcomas must be considered when evaluating congenital tumors, which grow rapidly despite their vascular appearance. Sometimes, they can be difficult to diagnose, requiring histopathological and immune-histochemical examinations. Surgical excision constitutes the therapeutic option of choice.

## Author contribution

Hafedh Daly, Faiez Boughanmi, Mohamed Ali Chaouch, Midani Touati, Fethi Jebali, and Bahri Mahjoub participated in the manuscript and validated the final version of the manuscript.

## Consent

Written informed consent was obtained from the patient's parent for publication of this case report and the accompanying images. A copy of written consent is available for review by the editor in chief of this journal upon request.

## Ethical approval

Ethical approval is exempt/waived at our institution, Monastir University Hospital, for all the case reports.

## Guarantor

Mohamed Ali Chaouch.

## Research registration number

N/A.

## Funding

This research did not receive grants from the public, commercial or not-for-profit sectors.

## Conflict of interest statement

No conflict of interest to disclose.
